# Editorial: Targeted genome editing for crop improvement

**DOI:** 10.3389/fpls.2023.1106996

**Published:** 2023-02-01

**Authors:** Maria Raffaella Ercolano, Kejian Wang

**Affiliations:** ^1^ Department of Agricultural Science, University of Naples Federico II, Portici, NA, Italy; ^2^ State Key Laboratory of Rice Biology, China National Rice Research Institute, Chinese Academy of Agricultural Sciences, Hangzhou, China

**Keywords:** CRISPR/Cas methodology, base editor (BE), prime editor, multiplex genome editing, agronomic traits

## Introduction

The genome editing approach is revolutionizing both agricultural and plant research. Conventional breeding strategies are often time-consuming and laborious, and may not be able to maintain the rate of progress requested with the increasing food demand (Scheben et al., 2017). There is a pressing need of new breeding techniques for developing agriculture products in sustainable way. In recent years, the targeted genome editing technologies showed that the precise modification of a trait is feasibly (Anzalone et al., 2020). Important technological developments, including optimization of clustered regularly interspaced short palindromic repeats (CRISPR)/CRISPR-associated protein 9 (CRISPR/Cas9) protocols in popular and neglected crops, and innovative methodologies approaches have been provided. In addition, several examples of traits improvement obtained through this methodology have already been made available (Zhu et al., 2020; Gao, 2021; Huang et al., 2021). This research topic presents the most recent advances in 19 publications, including 5 reviews, one method article and 13 research articles, contributed by 119 authors. The aim of this topic is to survey the major technological advances and application of genome editing in crops. Here we summarize these papers according the classification of contributions, mainly including the optimization of CRISPR/Cas systems in different crops and its applications in crops.

## Advancements in genome editing technology

The CRISPR/Cas9 system represents the third generation of targeted genome editing technology (Gaj et al., 2013; Gupta et al., 2019). The Zinc finger nucleases (ZFNs) and transcription activator-like effector nucleases (TALENs) has provided answers to basic questions related to plant biology as well as to compelling breeding needs. Although, the simplicity, the high efficiency and specificity of CRISPR/Cas9 allowed its rapid and widespread diffusion in plant science (Huang et al., 2022). This system has become increasingly mature and has been widely used in gene knockout, knock-in, and regulation, as well as the improvement of yield, quality, and biological resistance of important crops (Rao et al.). Several attempts for enlarging the opportunities offered by CRISPR/Cas9 system have been conducted. Engineering of Cas9, Cas12a, Cas12b, and Cas12f proteins was able to improve its efficiency (Mattiello et al.). The multiplex genome-editing (MGE) technologies allowed to enhanced mutations at multiple loci (Abdelrahman et al.). Recently, base editors (BEs) methodology displayed to be a powerful tool for altering desired trait in crops. Although the editing efficiency, and editing window are still not optimal, base substitution of target sequences by BEs can be accurately achieved. Moreover, prime editors (PEs) permitted to replace or insert sequences in crucial sites (Zhu and Zhu). In rice, although the fusion of a rice codon-optimized human Rad51 DNA-Binding Domain (DBD) protein between Cas9 nickase and the deaminase did not increase editing efficiency, the editing window of base editors was expanded. Similarly, the use of a specific rice Rad51 DBD homolog also expanded the editing window effectively (Wei et al.). Cytosyne and Adenine base editor (CBE and ABE) systems were successfully used to target the *SiALS* and *SiACC* genes in foxtail millet (*Setaria italica*). By utilizing CBE to target the *SiALS* gene, a homozygous herbicide-tolerant mutant was created (Liang et al.).

CRISPR/Cas systems targeting cell organelle genomes or RNA have also been explored and modified gene-editing systems made transgene-free plants more readily available. In various plant species, the hairy root induction system showed to be an effective method to study gene expression and function due to its fast-growing and high genetic stability (Jacobs and Martin, 2016; Gutierrez-Valdes et al.; Le et al.). An example hairy root induction mediated by *Rhizobium rhizogenes* for performing CRISPR/Cas9 editing was described in cucumber (*Cucumis sativus* L.). This system displayed to offer a wide range of possible applications to solve different challenges in cucumber as well as other cucumis plants (Van Nguyen et al.). The improvement of CRISPR-based technologies efficiency is enabling its implementation in a variety of crop plants, fostering the progress in both basic research and molecular breeding. Furthermore, the application of MGE and modified gene-editing technologies accelerated its use in crop-improvement programs.

## Breeding achievements

In less than a decade afterward, CRISPR/Cas-based technology has been successfully used as a powerful and efficient tool for genome editing due to its simplicity, efficiency, and versatility. In this research topics, the application of the CRISPR/Cas system involved many crops, such as rice, cucumber, oilseed rape, soybean, tomato, sweet orange, oil palm, alfalfa and tuber crops (Tussipkan and Manabayeva). The target engineered genes were found to be associated with nutrient–antinutrient content, post-harvest factors, abiotic-biotic resistance, self-incompatibility and recombination traits.

## Nutritional and post harvest traits

An interesting “proof-of-concept” approach was proposed in *Arabidopsis*, using ABE and CBE to obtain *FATTY ACID DESATURASE 2* (*FAD2*) alleles, whose functional alterations can reduce the unsaturation levels of fatty acids with acceptable plant growth defects. The authors claim that equivalent alleles may be generated in vegetable oil crops *via* precision genome editing for practical cultivation. Interestingly, all higher oleic-acid alleles turned out to arise within the g5 gRNA targeting region, which represents the cytosolic stretch of ER-membrane-bound FAD2 protein (Park et al.). An “trade-off” concept was presented in soybean using RNA interference to silence *CG-β-1* expression, which sharply raised the accumulation of 11S glycinin at the expense of reducing the content of 7S globulin (Wang et al.).

Vitamin E deficiency have a profound impact on human health. A daily supplement of vitamin E *via* high-quality rapeseed oil is the safest and most effective way to keep the nutritional requirement for the human body. Zhang et al. used CRISPR/Cas9 to perform targeted mutagenesis of *BnVTE4* homologs, the editing of which led to a significant change of the α-tocopherol content and the ratio between α- and γ-tocopherol, providing a theoretical basis for breeding high α-tocopherol content oilseed rape. Ascorbate is also an essential antioxidant substance for humans. The tomato (*Solanum lycopersicum*) gene *ASCORBATE PEROXIDASE 4* (*SlAPX4*), specifically induced during fruit ripening, is involved in the decrease of ascorbate. *SlAPX4* mutants, obtained by the CRISPR/Cas9 system, increased ascorbate content in ripened tomato fruits, but not in leaves (Do et al.). Those strategies supply novel formulas for food products.

In tomato, CRISPR/Cas9 system was also used to induce the targeted mutagenesis of the Polygalacturonase (PG) *SlPG* gene to delay the softening of tomato fruit. Mutated plants exhibited late fruit softening under natural conditions and lower water loss (Nie et al.).

## Developmental and environmental defense traits

Alfalfa mutated genotypes in *SQUAMOSA PROMOTER-BINDING PROTEIN-LIKE 8* (*MsSPL8)* genes using CRISPR/Cas9 technology displayed consistent morphological alterations, including reduced leaf size and early flowering. Plants with the highest number of mutated *MsSPL8* alleles exhibited significant decreases in internode length, plant height, shoot and root biomass, root length and drought tolerance (Singer et al.). In oil palm, the development of a transient protoplast assay and the generation of stable transformants allowed the CRISPR/Cas9 cleavage of *phytoene desaturase* (*EgPDS*) with good efficiency. The CRISPR/Cas9 system was further used to target the *brassinosteroid-insensitive 1* (*EgBRI1*) gene, which resulted in premature necrosis shoots and stunted phenotype mutants (Yeap et al.). Tomato mutant lines carrying targeted deletions of *Walls Are Thin 1* (*WAT1*) gene *SlWAT1* showed enhanced resistance to *Verticillium dahlia*, *Verticillium albo-atrum* and *Fusarium oxysporum* f. sp. *lycopersici* (*Fol*), but severe growth defects (Hanika et al.). A significant improvement to sweet orange genome editing was developed by choosing superior promoters [*Cestrum yellow leaf curling virus* (CmYLCV) or *Citrus sinensis* ubiquitin (CsUbi) promoter] to drive Cas9 and optimizing culture temperature. The author generated canker-resistant sweet orange by mutating the effector binding element (EBE) of canker susceptibility gene *CsLOB1*, which is required for *Xanthomonas citri* subsp. *citri* (*Xcc*) infection (Huang et al.).

Reactive oxygen species (ROS), which act as key regulators of anther development, are mediated by *Respiratory Burst Oxidase Homolog* (*RBOH*) genes. Knockout mutations by CRISPR/Cas9 of both *LeRBOH* and *LeRBOHE*, two tomato anther-expressed genes, resulted in complete male sterility. Further analysis of mutants provided helpful information for understanding how *RBOH* genes regulate tomato reproduction process (Dai et al.). Additionally, manipulation of the distribution and frequency of meiotic recombination events to increase genetic diversity and disrupting genetic interference is a hot-topic in crop breeding. Null mutants of the *ZEP1* gene, which encodes the central component of the meiotic synaptonemal complex (SC), produced male sterile mutants. Genetic recombination frequency was greatly increased and genetic interference was completely eliminated by crossing the *zep1* mutants with a male fertile variety. The remained female fertility of the *zep1* mutants makes it possible to break linkage drag. This study provides a potential approach to increase genetic diversity and fully eliminate genetic interference in rice breeding (Liu et al.).

## Perspectives

The Research Topic on *Targeted Genome Editing for Crop Improvement* collected innovative contributions on recent advances made in the field of plant GENOME EDITING. With the rapid development of genome editing technologies and functional genomics, it is foreseeable that many new optimized gene editing systems will emerge. Because of the limitation inherent in time constraints, this research topic did not cover the development, optimization, and application of gene editing systems for as many crops as possible. We hope that more original research and critical review papers on novel genome editing technologies such as CRISPR gene editing, MGE, base editing, and primer editing will be published in coming topics of Frontiers in Plant Science, helping to better guide the future research.

## Author contributions

ME and KW have made a substantial, direct, and intellectual contribution to the work, and approved it for publication in Frontiers in Plant Science.
